# High-Precision Phenotyping of Grape Bunch Architecture Using Fast 3D Sensor and Automation

**DOI:** 10.3390/s18030763

**Published:** 2018-03-02

**Authors:** Florian Rist, Katja Herzog, Jenny Mack, Robert Richter, Volker Steinhage, Reinhard Töpfer

**Affiliations:** 1 Julius Kühn-Institut, Federal Research Centre of Cultivated Plants, Institute for Grapevine Breeding Geilweilerhof, 76833 Siebeldingen, Germany; florian.rist@julius-kuehn.de (F.R.); robert.richter@julius-kuehn.de (R.R.); reinhard.toepfer@julius-kuehn.de (R.T.); 2 Institute of Computer Science 4, University of Bonn, Endenicher Allee 19 A, 53115 Bonn, Germany; mack@cs.uni-bonn.de (J.M.); steinhage@cs.uni-bonn.de (V.S.); 3 Institute of Crop Science and Resource Conservation (INRES)–Plant Breeding, University of Bonn, 53113 Bonn, Germany

**Keywords:** grapevine phenotyping, bunch compactness, sphere detection, Random-Sample-Consensus (RANSAC), Organization of Vine and Wine (OIV) descriptor 204, *Botrytis*, *Vitis vinifera*

## Abstract

Wine growers prefer cultivars with looser bunch architecture because of the decreased risk for bunch rot. As a consequence, grapevine breeders have to select seedlings and new cultivars with regard to appropriate bunch traits. Bunch architecture is a mosaic of different single traits which makes phenotyping labor-intensive and time-consuming. In the present study, a fast and high-precision phenotyping pipeline was developed. The optical sensor Artec Spider 3D scanner (Artec 3D, L-1466, Luxembourg) was used to generate dense 3D point clouds of grapevine bunches under lab conditions and an automated analysis software called 3D-Bunch-Tool was developed to extract different single 3D bunch traits, i.e., the number of berries, berry diameter, single berry volume, total volume of berries, convex hull volume of grapes, bunch width and bunch length. The method was validated on whole bunches of different grapevine cultivars and phenotypic variable breeding material. Reliable phenotypic data were obtained which show high significant correlations (up to r^2^ = 0.95 for berry number) compared to ground truth data. Moreover, it was shown that the Artec Spider can be used directly in the field where achieved data show comparable precision with regard to the lab application. This non-invasive and non-contact field application facilitates the first high-precision phenotyping pipeline based on 3D bunch traits in large plant sets.

## 1. Introduction

Grapevine (*Vitis vinifera* L. subsp. *vinifera*) is one of the most profitable crops worldwide. It is used for the production of wine grapes, table grapes and raisins [1]. Grapevine production is endangered from several fungal diseases (powdery mildew, downy mildew and *Botrytis*) that may cause severe economic losses. In particular, humid and warm conditions during the ripening period increase the risk for bunch rot infestations which are caused by the necrotrophic fungus *Botrytis cinerea* [2,3,4,5]. *Botrytis* can result in considerable yield and quality losses of grapes (a literature overview is given by Herzog et al. [6]). However, several studies have demonstrated that a dense bunch structure (i.e., bunch compactness) favors the infestation of grapes with *Botrytis* [2,3,4,5]. As a consequence, clonal selection and grapevine breeding focuses on the selection of genotypes revealing a loose bunch structure [6,7] which is known as one of the best strategies to increase resilience against *Botrytis* bunch rot.

However, bunch structure is defined by bunch architecture, a mosaic of single phenotypic traits. It is a composition of the number, the diameter and volume of berries in relation to the length, the width and the total bunch volume. The International Organization of Vine and Wine (OIV) published descriptors in order to rate and classify morphological grapevine traits visually [8]. With regard to *Botrytis* resilience, OIV descriptor 204 is commonly used in grapevine breeding and research in order to classify bunches at maturity, according to the level of compactness into five classes (class 1—very loose bunches up to class 9—very dense bunches). Currently, phenotyping requires several skilled employees, it is very time consuming and thus, the number of scored plants and repetitions are limited. Further, the remaining phenotypic data are subjective with unpredictable error variations and due to the 5-class classification system, single bunch traits cannot be recorded. In consequence, fast and reproducible phenotyping of the bunch architecture is absolutely required in order to ensure high-throughput phenotyping of objective, valid and high-precision data.

In recent years, sensor technology and sensor-based phenotyping approaches were therefore developed. The major aim is increased breeding/selection efficiency, based on an increased number of samples (high-throughput) and an improved objectivity of phenotypic data. Image-based analysis has been used often for the characterization of different bunch traits, like bunch length, width and bunch compactness [9,10,11,12]. Furthermore, it was shown that the number of berries and other berry characteristics, e.g., size and weight, could be extracted from images [13,14,15]. Three-dimensional (3D) methods enable the acquisition of the whole bunch architecture and the distribution of berries and the diameter of all visible berries. Thus, it is feasible to investigate bunches in its natural 3D structure saving time and labor. Until now, expensive 3D laser scanners [16] or imaging approaches [17,18,19] were developed to generate 3D point clouds in order to analyze bunch architecture related traits, i.e., bunch compactness, volume, length and width. All of these methods require expensive sensors (especially laser scanners) and/or complicated software for data analysis. This makes daily applications in grapevine research or breeding not feasible. With regard to this limitation, in the present study the fast, light, handheld and high-resolution 3D scanner of Artec Spider was established and an intuitive, user friendly and automated software was developed.

The software is an adapted modification of the algorithm developed by Mack et al. [20]. They employ Fast-Point-Feature Histograms as descriptor to classify 3D point clouds from grape bunches (acquired with fixed, high-resolving, near range laser scanner) into parts of “berries” or “stem”. Based on this information, they segment the berry points into regions and apply a RANSAC-based approach to fit sphere models into those regions to reconstruct the berries itself. From these models, properties like radii and volumes of the berries can be derived. 

The present study was divided into six major tasks: (1) establishment of Artec Spider 3D scanner to acquire dense 3D point clouds of grape bunches; (2) software development with an operator friendly graphical user interface (GUI) for automated analysis of 3D point clouds and extraction of parameters which are related to grape bunch architecture; (3) validation of extracted parameters with regard to precision and reliability as proof-of-principle on selected cultivars; (4) reliability test of the developed workflow for potential breeding purposes, i.e., phenotyping of breeding material with high varying bunch architecture characteristics (very loose up to very dense bunch structure); (5) calculation of factors with high correlation to OIV 204 based on 3D bunch traits; (6) the application of Artec Spider directly in the field as proof-of-principle for non-invasive and non-contact phenotyping field approach.

## 2. Materials and Methods

### 2.1. Sensor and Plant Material

**Figure 1:**
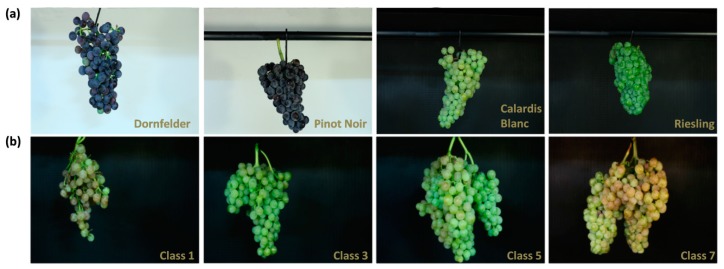
Grape bunches used in this study for data acquisition and parameter extraction. (**a**) Two red (Dornfelder, Pinot Noir) and two white (Calardis Blanc, Riesling) cultivars were used for validation of extracted bunch parameters and field application. (**b**) F1 progenies of GF.GA-47-42 × “Villard Blanc” divided into four different OIV 204 classes (class 1, 3, 5 and 7).

In this study the 3D scanner Artec Spider (Artec 3D, L-1466, Luxembourg,) was used. The sensor technology is based on blue LED structured light. The dense point clouds have a mesh resolution of up to 0.1 mm and point accuracy of up to 0.05 mm. Artec Spider is controlled by the Artec Studio 10 firmware. First, handling and accuracy of the 3D sensor was tested by the repeated scan of 10 table grape bunches of the cultivars Sultana, Sugraone, Sugarthirteen, Ruby Seedless, Prime (2 per cultivar). For further experiments, bunches were harvested at the experimental vineyard of Geilweilerhof in Siebeldingen, Germany, during the 2016 season according to the Biologische Bundesanstalt, Bundessortenamt und CHemische Industrie (BBCH) scale (https://ojs.openagrar.de/index.php/BBCH/article/view/483/433) at the developmental stages BBCH 87–89 (BBCH89 = berries ripe for harvest). In order to test the transferability of the novel method as proof-of-principle, 39 dense bunches (Riesling as white and Pinot noir as red cultivar) and 35 looser bunches (Calardis blanc as white and Dornfelder as red cultivar) were scanned. The selected cultivars show high variability in berry color, bunch/berry size, shape, and bunch compactness (Figure 1a). Further, robustness and reliability of received 3D bunch data were tested by applying the phenotyping pipeline on the highly variable F1 progeny of the crossing population of GF.GA-47-42 × “Villard Blanc” [21] (Figure 1b). Therefore, 222 bunches of 41 genotypes (at least three bunches per genotype, Supplementary Table S1) were used. Finally, 48 grapes of Dornfelder, Pinot Noir, Calardis Blanc and Riesling (12 per cultivar, BBCH 89) were scanned directly in the field and further, under standardized lab conditions with the aim to validate precision and reliability of the received phenotypic data under field conditions.

### 2.2. 3D Data Acquisition

**Figure 2:**
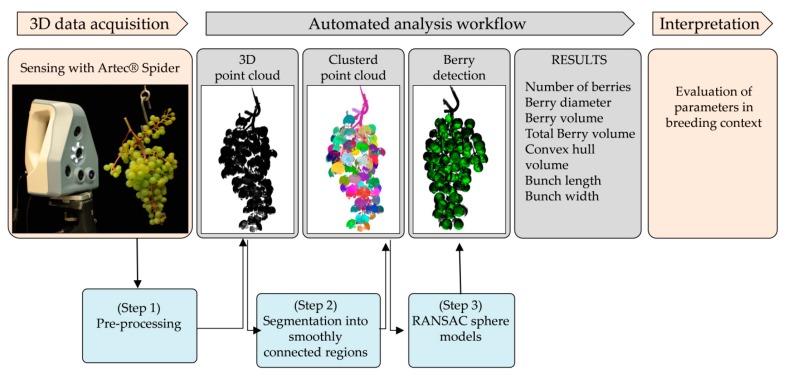
Phenotyping pipeline: data acquisition and data analysis to phenotype 3D bunch traits. Bunches were fixed on a motorized hook and scanned 360° (or from the front) resulting in a dense point cloud of the bunch. Data analysis was conducted with 3D-Bunch-Tool (3D-BT). In a first step, the point cloud is segmented and characterized in its visible parameters. The output data are number of berries, berry diameter, berry volume, bunch length, bunch width and convex hull volume.

Under lab conditions (standard ceiling illumination and room temperature 20 °C), grape bunches were fixed on a hook and scanned from the visible side (partial scan). For phenotyping the entire bunch architecture, bunches were hooked on a motorized device with controllable rotation speed and the whole bunch was scanned (360° scan) with up to 7.5 frames per second. During spinning, the scanner recorded geometry and color data of the grape and transformed it into detailed 3D point clouds (Figure 2). Data were saved for further analysis in Polygon File format (PLY). 

### 2.3. “3D-Bunch-Tool” with Graphical User Interface

The 3D-Bunch-Tool (3D-BT) is a modified algorithm as described by [20] and consists of a three-step workflow as shown in Figure 2:

Step (1) Pre-processing step: Reduction of high-resolution point cloud to reduce computing time.
Step (2) Segmentation step: all points of the point cloud are segmented into smoothly connected regions using a region growing approach (Figure 2). Most of these regions contain one berry, but due to irregularities and occlusion in the data, it is possible that more than one berry is included in a region (undersegmentation) or a berry is split into several regions (oversegmentation).
Step (3) Berry detection step: We use a RANSAC-based approach to fit sphere models into the data, taking care of undersegmentation by extracting the inliers for each sphere from the region and reusing the remaining data until the number of points contained in the region fall below the minimal number of inliers or no model could be found (Figure 2). Only sphere models showing a radius in the range between minimal and maximal berry radius and a sufficient number of inliers, i.e., points lying close to the surface of the model, are kept. A post-processing step is used to deal with oversegmentation: all sphere models with significant overlap (more than 25%) are compared to each other and only the one with the most inliers is considered to be a detected berry.

**Figure 3:**
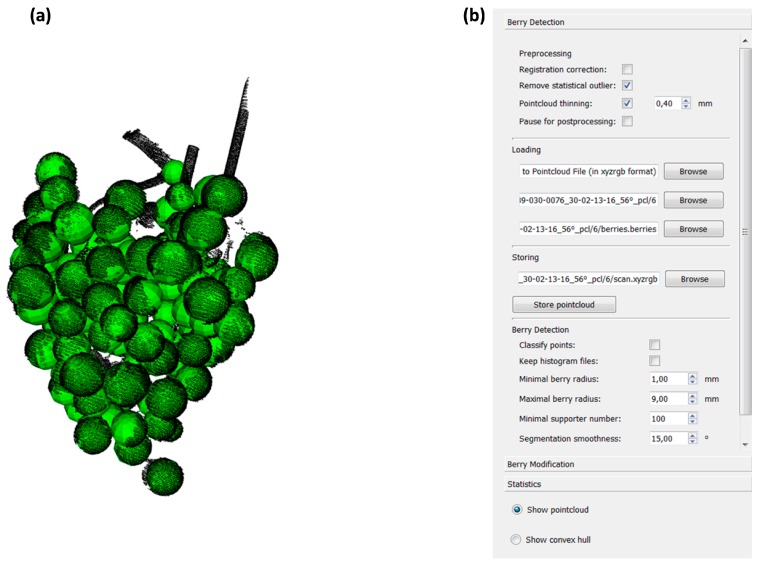
Graphical User Interface (GUI) of 3D-Bunch-Tool (3D-BT). The viewport of grape bunch reconstruction is shown. Detected berries are shown as green spheres (**a**). The settings field is located on the right side of the GUI with adjustable parameters (**b**).

Finally, an intuitive Graphical User Interface (GUI) was developed consisting of a viewport (left) and a settings field (right) in order to provide easy access and fast overview over the berry detection process (Figure 3).

After import of a 3D point cloud, the current state of berry detection, the segmented cloud (every region shown in a different color as visible in Figure 2—“Clustered point cloud”) to the finally reconstructed berries, represented as spheres (Figure 2—“Berry detection”) is shown on the viewport. The settings field shows the current parameters and provides the possibility to adjust them if necessary (Figure 3). We used parameters slightly adjusted from [20], as the minimal and maximal berry radius available in the data is greater (between 1 mm and 9 mm, respectively) and the point density achieved with the Artec Spider Scanner higher, therefore the minimal supporter number had to be set to a different value (100) and we are able to use a larger resolution of 0.4 mm. For fast processing of a high number of point clouds, we provide an option to select a folder including several scans in PLY format. In this mode, the processing workflow is automatically applied to all point clouds using the current settings and the detected berries and their respective output data are stored. As shown in [20], few erroneous sphere models can remain after the post-processing step in critical areas, like the hook. While the GUI provides the possibility to manually remove such erroneously detected spheres, we can expect that they will only lead to a minimal discrepancy in the statistically generated output data. Therefore, we decided not to include this manual and therefore time consuming step. 

Finally, the software exports the following 3D bunch traits (.txt):

(1) Maximal bunch length and bunch width, i.e., maximal diameter of the grape bunch parallel to the y-axis (length) and the maximal diameter parallel to x- or z-axis (width).
(2) Volume of the convex hull of all points lying inside a detected berry.
(3) Average diameter of the detected berries, i.e., average berry size.
(4) Average volume of the detected berries.
(5) Total berry volume.

### 2.4. Ground Truth Data and Statistics

Objective ground truth data of bunch phenotypes were acquired by using established image-based methods as described by [13,22]. Therefore, images were captured from the front side of the bunch, every single berry from the bunch was removed and berries were manually distributed on a perforated plate and a second image was captured [13]. The images were automatically analyzed with MATLAB based tools [13,22]: length and width of grapes were determined by the Trait-Size-Tool (TST) [22], the number of berries, berry size and berry volumes were acquired by the Berry-Analysis-Tool (BAT; [13]). Statistical analysis was conducted with R (Version 3.4.1). One-way-ANOVA analysis with Duncan multiple range test, Pearson correlation coefficient and for factor analysis, Spearman’s rank correlation coefficient were implemented in order to validate 3D bunch traits with ground truth data.

## 3. Results and Discussion

### 3.1. Establishment of Artec Spider 3D Scanner

Grapevine bunches differ widely in shape and form from their phenotype. First of all, the Artec Spider 3D scanner was used to scan 10 table grapes 10 times respectively with different rotation speeds [five times fast (0.5 s^−1^) and five times slow (0.16 s^−1^)]. The different rotation speeds result in point clouds with different point densities. The point clouds were analyzed with 3D-BT. Obtained results showed no significant differences between the investigated scanning variants. Thus, we assumed that the Artec Spider provides valid data for reliable characterization of bunch architecture determining traits (Table 1).

### 3.2. Proof-of-Principle on Selected Grapevine Cultivars

**Figure 4:**
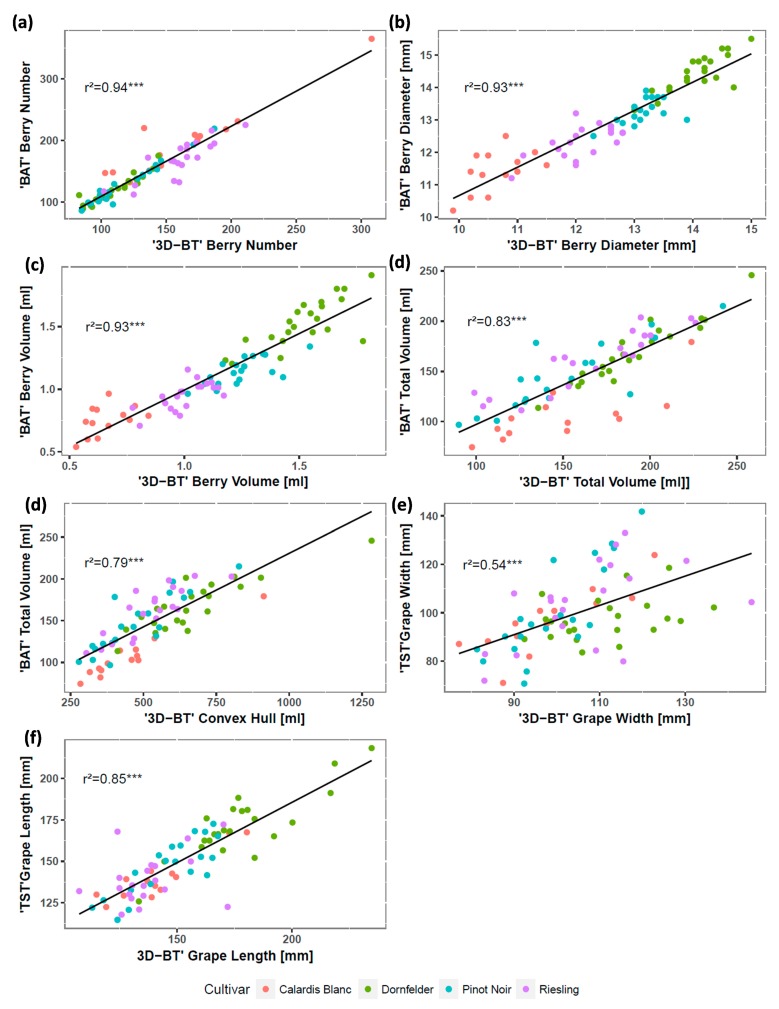
Experimental correlation plots of 3D bunch traits (3D-BT) in comparison to ground truth data (BAT/TST) for number of berries (**a**), berry diameter (**b**), berry volume (**c**), total berry volume (**d**), convex hull/total berry volume (**e**), grape width (**f**) and grape length (**g**). Cultivars are colored in red (Calardis Blanc), green (Dornfelder), blue (Pinot Noir) and purple (Riesling), *** *p* < 0.001. BAT—Berry-Analysis-Tool; TST—Trait-Size-Tool.

In the next step, the application of the phenotyping pipeline was tested on four selected grapevine cultivars. Received 3D bunch traits were correlated to ground truth data. Correlation plot based on all phenotypic data is shown in Figure 4 (bunch structure- and cultivar-specific results are given in Supplementary Table S2). 

The results showed that berry number, berry diameter and the berry volume achieved very high correlation coefficient values. Correlation values for the total berry volume (Figure 4d, r^2^ = 0.83, *p* < 0.001) can be explained by the summation of the 3D-BT underestimation of the berry number and its related berry volume. Convex Hull showed r^2^ value of 0.79 (*p* < 0.001) compared to the total berry volume, measured with the image-based BAT (Figure 4e). The convex hull regards the volume of the whole bunch. That means that not just the area taken by berries but also the area between the berries and the area which is taken by the skeleton structure is considered. Therefore, the value for the convex hull will always be higher compared to the total berry volume. Moreover, it can be assumed that values for bunches with a big secondary bunch or a high number of interior berries, will lead to increasing values for the convex hull compared with BAT total berry volume. For the bunch width, r^2^-value was the lowest (r^2^ = 0.54, *p* < 0.001) compared to 2D based TST reference measurements (Figure 4f). From a geometrical point of view, TST calculates grape bunch width as the longest distance between two berries with respect to x- and y-coordinate. 3D-BT extended this three dimensional and can also detect berries among the z-axis to take distance measurements. Thus, single measurement values vary stronger between the 2D and the 3D method. According to OIV 202, the length of the bunch is measured between the first and the last berry of the bunch in a vertical way. Adapted on OIV 202, the algorithm of 3D-BT detects the two most distant berries vertically and estimates the distance between them. For the bunch length correlation coefficient of 0.84 with corresponding *p* < 0.001, was detected compared to measurements taken with the TST 2D tool. (Figure 4g). Data analysis using BAT and TST requires lot of manual work, e.g., removal of every single berry from the bunch, manual distribution of all berries on a perforated plate [13]. According to the size of the bunch and the total number of berries, the whole procedure needs on average 10 min per bunch. Thus, it is very labor-intensive and invasive. In comparison, acquisition of point clouds by using the Artec Spider and data storage, need on average one minute and therefore this represents an up to 10-times faster, robust and non-invasive method. In addition, it enables the investigation of the bunch architecture in its natural 3D structure. In summary, obtained 3D bunch traits show slight differences on a cultivar /bunch structure level (Supplementary Table S2), i.e., 40 out of 42 calculated correlations are significant. Detected differences between received mean 3D bunch traits in comparison to mean ground truth data were also small (except for convex hull), e.g., mean berry diameter differs on average by 0.36 mm or mean berry number differs on average by 15 berries. The results indicate that precision and correlation values, especially the number of berries or berry diameter/volume, are more affected by the whole bunch architecture (sum of berry and bunch parameter as cultivar characteristics) as by structure of investigated bunches (loose- in comparison to dense bunch structure). In conclusion, the developed phenotyping pipeline (Figure 2) and precision of obtained 3D-BT phenotypic data are valid and reliable with regard to potential applications, i.e., breeding purposes and (breeding) research.

### 3.3. Test of Reliability: Application of the Workflow on High Varying Breeding Material

For breeding purposes, the developed pipeline must be robust enough to phenotype high varying breeding material, i.e., seedlings, progenies and grapevine accessions with different bunch characteristics (e.g., very loose up to very dense architecture, different bunch volume, berry size as well as different berry number). Therefore, segregating crossing progeny of GF.GA-47-42 × “Villard Blanc” [21] was used to test the efficiency of the phenotyping pipeline. Calculated correlations (Table 2) were very high (except for the traits of grape width/length). Differences were observed between 3D-BT and ground truth data with the number of detected berries (Table 2). This can be explained due to the amount of inner layer berries of compact bunches. These inner components are not externally visible and therefore cannot be detected non-invasively. However, the correlation coefficient was very high (r^2^ = 0.95, *p* < 0.001) (Table 2). Correlation coefficient values (Table 2) were also comparable to results given in Figure 4 and Supplementary Table S2, although berry diameter is slightly different. Higher correlations could be explained by higher number and variability of investigated bunches, e.g., berry sizes range from 9.2–16.9 mm (Figure 4: 10.2–15.5 mm). 

Convex hull and total berry volume (Table 2) showed similar r^2^-values compared to the results achieved in Section 3.2. Lower correlation values were observed for the grape width and grape length (r^2^ = 0.59–0.57, *p* < 0.001) (Table 2). As observed on selected cultivars (Figure 4), 3D-BT overestimates the bunch width/length in comparison to ground truth data. Overestimation means a deviation of the average 5.2 mm for grape width and the average 23.9 mm for grape length. Contrary to the investigated bunches in Section 3.2, bunches of segregating F1 progeny contain frequently large secondary bunches and bunch phenotypes vary strongly from genotype to genotype. However, the results from Section 3.2 and Section 3.3 indicate that the phenotyping pipeline is robust and expedient for valid bunch trait characterization of breeding material.

### 3.4. Factor Analysis for an Objective Assessment of Bunch Compactness

**Figure 5:**
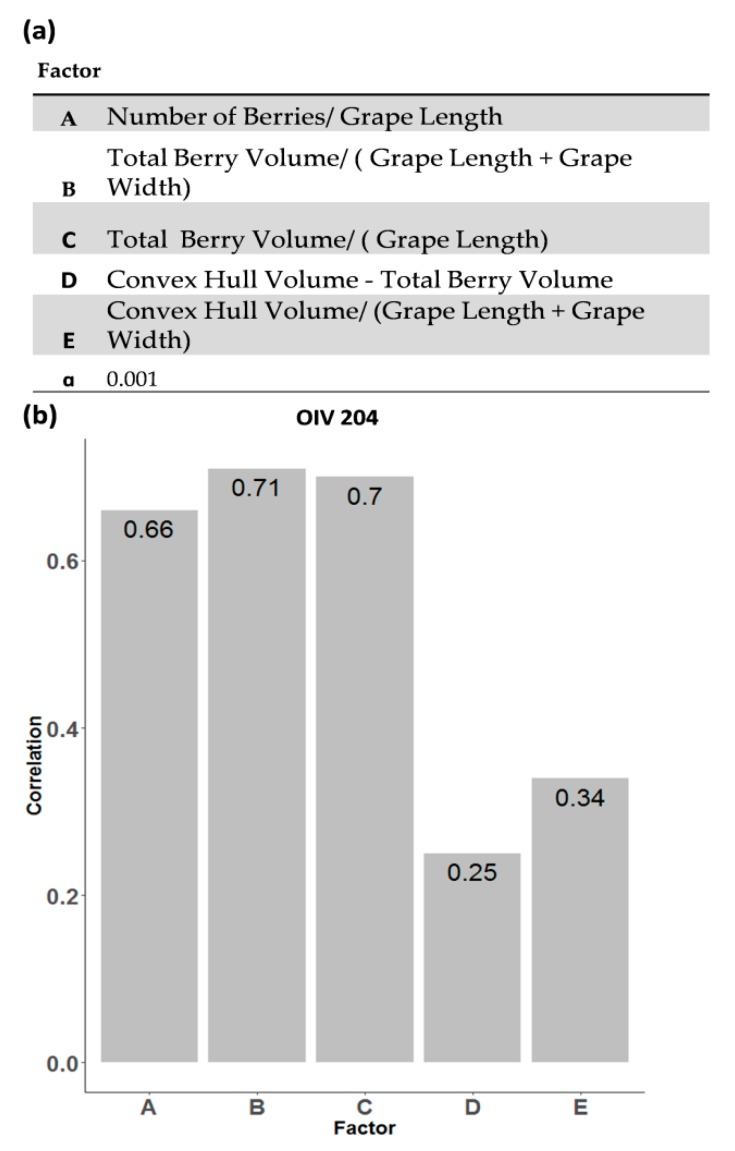
Compactness factors for objective evaluation of OIV 204 descriptor. (**a**) Five factors were estimated from the traits extracted from 3D sensor data (**b**). Correlation values of the factors with OIV 204 descriptor classification. n = 100.

Based on the findings of Pommer et al. [23] and previous experiments (unpublished), correlations between bunch compactness and bunch volume, number of berries and berry volume were detected. Thus, in the present study five quantitative factors were tested for an objective assessment of bunch compactness (Figure 5a). A subset of 100 bunches from the F1 progeny were analyzed and compactness was classified according to the optical descriptor OIV 204 [8]. The samples represent the OIV 204 classes 1–7. The factor values were correlated with OIV 204 classification (Figure 5).

The highest correlation values were observed for Factor B and Factor C (0.71–0.7, *p* < 0.001) (Figure 5b). Those factors consider the total berry volume, grape length and further grape width (Figure 5b). Factor A [23] achieved a slightly lower value (0.66, *p* < 0.001). Factor D and E showed the lowest correlation values (0.25–0.34, *p* < 0.001) including the convex hull volume of bunches (Figure 5a,b). This result indicates that the 3D convex hull volume seems to be inexact for representing the volume of the whole bunch structure. This includes the total berry volume but also the volume of the empty space between the berries, which increases in loose and decreases in compact bunches. The result indicates that bunch architecture parameters like total berry volume, grape length and grape width might play an important role in bunch compactness. Moreover, the 3D bunch traits and the calculation of Factor B is usable for an objective, non-destructive assessment of bunch compactness with high-precision and high-throughput and provides a valid basis for further studies.

### 3.5. Proof-of-Principle: Field Application Test for Non-Invasive, High-Precision Phenotyping

**Figure 6:**
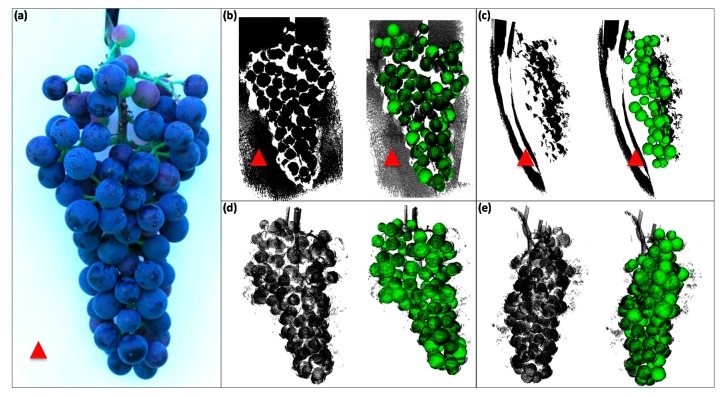
Application of the phenotyping pipeline in the field. Grape bunch of cultivar Dornfelder was prepared for scanning with artificial background (**a**). Front view of raw (left) and analyzed (right) 3D point cloud (**b**). Side view of the raw (left) and analyzed (right) 3D point cloud (**c**). Front view of the corresponding 360° scan from the point cloud in the lab. Raw (left) and analyzed (right) (**d**) 3D point cloud. Side view of the corresponding 360° point cloud. Raw (left) and analyzed (right) 3D point cloud (**e**). Red arrow heads mark the artificial background of partial point clouds.

Applications of the Artec Spider to scan bunches directly in the field facilitates the opportunity to acquire 3D bunch traits non-invasively and fast. The major challenge is the limited view on bunches; i.e., only partial scans are possible under field conditions resulting in incomplete 3D point clouds with lack of information between artificial background and visual side of the bunch (illustrated in Figure 6).

Randomly chosen sides of 10 table grape bunches were scanned 10 times in the lab (partial scan). 3D bunch traits were compared to the results of full scans (360° scan). 3D bunch traits based on partial scans (Table 3) show high, significant correlations to those received from 360° scans. Partial scans reveal only half berry numbers and thus, half total volume/convex hull in comparison to the phenotypes acquired from 360° scans. If traits like grape width/length were determined from partial scans, determined phenotypic data can differ more or less slightly, i.e., on average 15 mm for grape width and 12.7 mm for grape length (Table 3). One explanation might be the fact that geometry of bunches differs between varying perspectives and thus grape length/width depend on scanned side. Finally, these results were rated as reliable enough to assume that non-invasive, partial scans of grapes in the field are promising for contactless, fast and precise phenotyping of grape bunch architecture.

For that reason, the sensor was applied to scan grape bunches non-invasively in the field with three major objectives: (1) Sensor test under field conditions; (2) Test of automated program 3D-BT by analyzing 3D point clouds of incomplete, partial scanned grapes; and (3) Precision of 3D bunch traits derived from partial scans and corresponding 360° scans. Therefore, 48 bunches of the selected cultivars Dornfelder, Pinot Noir, Calardis Blanc and Riesling (12 represent bunches per cultivar) were scanned in the field contact-free and non-invasive (field scan). Sensor handling and acquisition of 3D point clouds was comparably easy to apply under lab conditions. One challenge was scanning during windy conditions because of slightly moving vines and bunches. This can be lead to an interruption or complete loss of the scanner’s tracking process. In order to prevent signal loss due to airstream, artificial background was used. Prior to the scanning process, interfering leaves were removed and an artificial background was used to avoid the overlap of adjacent bunches. Afterwards, scanned bunches were harvested and partial and full scans (360°) were conducted in lab as reference (an overview is given in Supplementary Table S3). 

Table 4 shows statistics of the three types of scans. The number of berries showed significant differences between 360° and partial/field scans but not between the partial scan in the lab and the field scan. Berry numbers of partial and field scans were approximately half of the total berry number obtained from 360° scans (Table 4). The calculated values for the parameters berry diameter and volume showed no significant differences between all three types of scans (Table 4). Total berry volume of the field-, front scan was slightly more than half compared to 360° values and as expected with statistically significant differences compared to 360° scans (Table 4). Strong significant differences were observed for the convex hull volume between all three methods (Table 4). This is a result of the fact that the convex hull is calculated by the connection of the most outer berry points, which are detected in the point cloud. This can differ widely with respect to the scanned side of the bunch as described previously. However, deviation of determined grape width based on field scans in comparison to 360° scan is only 7.2 mm.

The results show that 3D scans under field conditions provide high precision and reliable point clouds to determine different 3D bunch traits. The data can also be used to extrapolate total number of berries and thus, total berry volume. 

## 4. Conclusions

Grape bunch architecture, which defines bunch structure, relies on a mosaic of different single traits which could be acquired with the developed phenotyping pipeline in an objective, accurate and high-throughput manner. The phenotyping pipeline is open to all kind of users due to simple-to-handle unharmed sensor technology, analysis software with an intuitive graphical user interface and further, minor necessity of user interaction due to automated data analysis. These are the most convenient advantages of the developed method. Furthermore, acquisition and analysis of sensor data takes approximately one minute and is thus much faster compared to comparable precise methods [13]. Further, field applications can be used for repeatable screenings and comparable evaluations of large experimental plots of high varying breeding material or genetic repositories, e.g., in order to conduct comparative genetic association studies or to develop genetic markers for marker-assisted selection. This kind of phenotypic objectivity enables monitoring purposes in order to track bunch development under different environmental conditions, e.g., soil composition, water and nutrient availability.

## Figures and Tables

**Table 1:** Comparison between fast (0.5 s^−1^) and slow (0.16 s^−1^) scanning method. *p*-values from the One-way-ANOVA results for the 3D-BT estimated traits number of berries, berry diameter, berry volume, total volume convex hull volume (Convex Hull), grape width and grape length. Different letters indicate significant differences.

Method	Number of Berries	Berry Diameter [mm]	Berry Volume [mL]	Total Volume [mL]	Convex Hull [mL]	Grape Width [mm]	Grape Length [mm]	α
fast	72 ^A^	17.2 ^A^	2.7 ^A^	191.9 ^A^	948.8 ^A^	123.3 ^A^	176.9 ^A^	0.001
slow	70 ^A^	17.1 ^A^	2.7 ^A^	185.7 ^A^	929.6 ^A^	123.2 ^A^	176.6 ^A^	0.001

**Table 2:** Correlation Coefficient with related *p*-values and min, mean, max values for bunch parameters of genotypes from the cross population GF.GA-47-42 × “Villard Blanc”.

Method:	3D-BT					Ground Truth		
>**Trait**	**Min**	**Mean**	**Max**	***p*-Value**	**r^2^**	**Min**	**Mean**	**Max**
Number of Berries	12	128.5	322	<0.001	0.95	11	163	491
Berry Diameter [mm]	9.2	13.2	16.9	<0.001	0.87	7.6	13.8	17.4
Berry Volume [mL]	0.5	1.54	2.54	<0.001	0.9	0.2	1.37	2.79
Total Berry Volume [mL]	19	212	504	<0.001	0.95	2	235.7	678
Convex Hull/Total Berry Volume [mL]	129	770	3069	<0.001	0.81	2	235.7	678
Grape Width [mm]	61.82	123.32	199.28	<0.001	0.59	58.9	118.1	170
Grape Length [mm]	106	166.1	277	<0.001	0.57	66.7	142.2	261.9
n = 222								

**Table 3:** Correlation Coefficient r^2^ and significance (*p*-values) and mean values of seven 3D bunch traits between partial and 360° scan.

Method	Partial vs. 360°			
	Correlation Analysis		Mean Values	
**3D Bunch Trait**	**r^2^**	***p*-Value**	**Partial**	**360°**
Number of Berries	0.83	<0.001	37	71
Berry diameter [mm]	0.78	<0.001	17.1	17.1
Berry volume [mL]	0.8	<0.001	2.6	2.7
Total Berry Volume [mL]	0.82	<0.001	97.6	188.8
Convex Hull [mL]	0.72	<0.001	431	939
Grape Width [mm]	0.71	<0.001	108.5	123.3
Grape Length [mm]	0.7	<0.001	165	176.7
n = 100				

**Table 4:** Comparison of 3D bunch traits obtained from three different types of grape scans: fast and non-invasive field scans vs. invasive partial/360° scans in the lab. Different letters indicate significant differences. One-way-ANOVA with α < 0.001. n = 48.

Type of Scan	Number of Berries	Berry Diameter [mm]	Berry Volume [mL]	Total Volume [mL]	Convex Hull	Grape Width [mm]	Grape Length [mm]
360°	137 ^A^	12.8 ^A^	1.2 ^A^	152.6 ^A^	574.8 ^A^	106.4 ^A^	159.1 ^A^
partial	66 ^B^	13.1 ^A^	1.3 ^A^	79.7 ^B^	290 ^B^	90.5 ^B^	151.9 ^A^
field	66 ^B^	13.3 ^A^	1.3 ^A^	84.9 ^B^	409.2 ^C^	109.6 ^A^	155.8 ^A^

## Supplementary Materials

The supplementary materials are available online at http://www.mdpi.com/1424-8220/18/3/763/s1.
